# Editorial: Next generation bioanalysis by microfluidics-integrated methods, devices, and systems

**DOI:** 10.3389/fbioe.2025.1535365

**Published:** 2025-01-23

**Authors:** Jian Zhang, Jie Jayne Wu, Xiaonan Yang

**Affiliations:** ^1^ College of Electrical and Electronic Engineering, Wenzhou University, Wenzhou, China; ^2^ Department of Electrical Engineering and Computer Science, The University of Tennessee, Knoxville, TN, United States; ^3^ School of Electrical and Information Engineering, Zhengzhou University, Zhengzhou, China

**Keywords:** bioanalysis, integrated microfluidics, device, sensor, method, system

Owing to the vital role in disease diagnosis and health monitoring, bioanalysis has attracted more and more attention in recent decades. Sensitive, rapid-responding, high-throughput, and field-deployable bioanalysis has shown great advantages and prospects. Microfluidics-integrated methods, devices, and systems will promote the development of next generation bioanalysis by their powerful performance of particle manipulation.

The goal of this Research Topic is to provide a forum sharing recent research and novel ideas on related techniques. Five articles have been collected, including three research papers and two reviews. The techniques include microfluidic structures, microfluidic effects and the sorting/enriching methods.

For research papers, Zhang et al. present the improved constrictional microchannels for restricting single-cells to analyze their targeted proteins using fluorescent flow cytometry combined with recurrent neural network method. The sale of the microchannels is designed and fabricated to avoid the channel blockage with lower throughputs. In this work, multiple techniques are employed to identify and classify the single-cell targeted proteins, including fluorescent-flow recognition and neural network classification. However, both mentioned techniques should based on the first-step microfluidic cell manipulation to ensure a single-cell analyte with a high throughput. Therefore, the integrated microfluidics becomes a precondition in this work. The application of special microchannels illustrates the great advantage of microfluidics in single-cell manipulation.

Li et al. realize a transendothelial electrical resistance measurement with a microfluidic device for endothelial barrier assessment in inflammatory bowel diseases (IBDs). In this work, the authors use a well-designed microfluidic chip integrated with four electrodes independent from the outlets to detect the function of the endothelial cellular barrier after treatment with exosomes from the serum of IBD patients. This chip comprises two layers of microfluidic chambers with the top layer for medium perfusion maintaining the nutrition and pressure during cell culture, and the bottom layer for extracellular matrix mimic, which are separated by a semipermeable membrane permitting the formation of endothelial cell barrier. This system works as an organ chip, providing an effective platform for *in vitro* cellular analysis and demonstrating a prospect of microfluidics for physiological environment construction on chips.

Bi et al. report a fast-responding immunosensor for circulating tumor antigen for breast cancer employing alternating current electrokinetic (ACEK) effect and capacitive sensing method. ACEK refers to micro-flows or embedded particles moving directly towards the electrode surface under a certain AC signal in a solution with certain conductivity (Lian and Wu, 2009). It is an efficient microfluidic enrichment strategy without any additional microfluidic devices. This sensor with a detection limit of 1 ng/mL and four orders of concentration magnitude is qualified for breast cancer biomarker determination. With similar strategy, lower detection limits, wider dynamic ranges for various biotargets are also reported (Zhang, et al., 2024; Qi et al., 2022; Zhang et al., 2020), indicating a good adaptability of ACEK effect for sensitive and fast-responding biosensors.

For reviews, Peng et al. introduce the latest developments on sheathless inertial particle focusing methods. As passive focusing strategies, sheathless inertial focusing techniques demonstrate convenience and low cost maintaining strong performance. In this review, principles for hydrodynamic forces is first induced. Sheathless inertial microfluidic focusing methods are then summarized, including particle focusing in straight/curved microchannels, micro-vortex-induced particle focusing, and particle focusing in multi-staged microchannels. As a competitive microfluidic route, precise and high-throughput sheathless inertial particle focusing plays a prerequisite role for cell counting, biomolecular detection, sample sorting, and so on.

Tang et al. provide an integrated review on Raman cell sorting techniques for single-cell phenotyping and genotyping. The authors first introduce common Raman cell sorting methods, including Raman-activated microfluidic sorting, Raman-tweezer cell sorting, Raman-activated droplet sorting, etc. The mentioned three techniques are all with microfluidic component or method, which are microflow channels and dielectrophoresis (DEP)-based droplet sorting. Further, methods for Raman signal enhancement are summarized, including acoustic hydrodynamic focusing, flow cytometry, DEP force, hydrodynamic focusing and acoustofluidic focusing. According to this review, microfluidics in fact exists in almost all Raman cell sorting methods, including the structures and acoustic/electrical stimulation for cell diverting, focusing and enriching.

In summary, the papers collected in this Research Topic relate to several microfluidic fields in single-cell analysis, biological barrier simulation, microfluidic biosensor and sheathless inertial particle focusing. When microfluidics was employed to bioanalysis as key techniques, a consensus was formed that it would greatly promote the development of advanced bioanalytical platforms. It is foreseeable that the next generation bioanalysis will become more precious, more fast, more high-throughput, more affordable, more miniaturized, and even more intelligent with the integrated microfluidic methods, devices, and systems.
